# Molecular Characterization of Viruses from Clinical Respiratory Samples Producing Unidentified Cytopathic Effects in Cell Culture

**DOI:** 10.3390/v1020084

**Published:** 2009-07-17

**Authors:** Yacine Abed, Guy Boivin

**Affiliations:** Research Center in Infectious Diseases, CHUQ-CHUL and Laval University, Quebec City, QC, Canada; E-mail: Yacine.Abed@crchul.ulaval.ca (Y.A.)

**Keywords:** SISPA, respiratory viruses, picornaviruses, cardioviruses

## Abstract

The sequence-independent single primer amplification (SISPA) method was performed to identify a virus in 17 clinical respiratory samples producing uncharacterized cytopathic effects in LLC-MK2 cells. Sequence analysis of 600–1600 bp amplicons allowed the identification of six viruses (one influenza C, two parechovirus-3 and three cardioviruses). Genomic sequences of the cardioviruses showed similarities with those of the recently-described Saffold virus strain although significant variation was present in the viral surface EF and CD loops. These results demonstrate the usefulness of SISPA for identifying emerging viruses and also known viruses not easily identified by standard virological methods.

## Introduction

Respiratory viral infections (RVIs) are a major cause of morbidity and mortality worldwide. RVIs can be associated with a variety of clinical symptoms ranging from self-limited infections to more devastating conditions such as pneumonia [1]. Despite the use of a variety of laboratory diagnostic methods, in ≈ 50% of cases of community-acquired pneumonia in adults and in 15–35% of cases of bronchiolitis and pneumonia in children an etiologic agent still cannot be identified [2, 3]. It is likely that still unidentified viruses, as well as those that cannot be easily detected by current diagnostic methodologies, are responsible for the vast majority of unknown respiratory syndromes.

Each year, an average of 7000 clinical samples are sent to the Regional Virology Laboratory of Québec City (Province of Québec, Canada) for viral identification using antigen detection tests, cell-culture based techniques and PCR/RT-PCR assays. Interestingly, for a few samples, viral identification could not be achieved despite the presence of cytopathic effects (CPE) that could be observed in one or more cell lines. The aim of this study was to assess the ability of the sequence-independent single primer amplication (SISPA) method to identify some viruses producing uncharacterized CPE. This method, previously described in 2001 by Allander and colleagues [4], demonstrated efficacy in identifying known and new DNA and RNA viruses from clinical samples [4–6].

## Results and Discussion

Among the 17 clinical respiratory samples tested, the SISPA method allowed the identification of six viruses (Table 1). The latter viruses originated from four nasophayngeal aspirates (NPA) and two bronchoalveolar lavages (BAL). The identified RNA viruses consisted of influenza C virus which belongs to the *Orthomyxoviridae* family (one case), human parechovirus type 3 (HPeV-3) which belongs to the *Picornaviridae* family (parechovirus genus) (two cases) and cardiovirus Saffold-like virus which belongs to the *Picornaviridae* family (cardiovirus genus) (three cases). All 17 samples gave positive CPE when cultured on LLC-MK2 cells. The Canadian cardiovirus Can112051-2006 also presented CPE on Hep2 cells. The mean time to first CPE detection in LLC-MK2 cells was 19, 6 and 4 days for influenza C, HPeV-3 and cardioviruses, respectively (Table 1). The absence of commercially-available monoclonal antibodies and/or the novelty of these viruses precluded their rapid identification in the clinical virology laboratory. For instance, influenza C viruses display a poor growth in cell culture and are not typically identified by DFA or ELISA assays [7]. HPeV-1 and -2 (formerly echoviruses 22 and 23) have been known for years [8]. Recently, a variety of new HPeV types have been described [8]. RT-PCR assays based on the amplification of 5′ untranslating region (UTR) of enteroviruses will not detect parechoviruses because of significant genome sequence differences between these two viral genera [9]. Previous studies demonstrated that most HPeV-3 infections were associated with cases of neonatal sepsis [10, 11]. By contrast, the two HPeV-3 infections from this study were both associated with pneumonia, one in a 37-year old immunocompromised patient and the other in a 61-year old immunocompetent patient. Of note, the BAL of both patients also grew cytomegalovirus. Thus, the exact contribution of HPeV-3 infections in these two clinical cases remains unclear.

The Saffold virus, a recently identified cardiovirus, was initially isolated from the stool of a 8-month old girl who presented with fever of unknown origin [6]. The complete genomic sequence of this strain (OSV) was subsequently published in GenBank (accession number EF165067). Our three Saffold cardiovirus strains were recovered from NPA samples of children including a 23 month-old patient who was hospitalized for respiratory symptoms (fever, cough, rhinorrhea, otitis) (case 1), a 19 month-old patient who was hospitalized for a suspicion of bacteriemia and cold (case 2) and a 48 month-old patient who was hospitalized for pneumonia and otitis (case 3). The three Canadian strains were closely related based on partial sequences of the VP1 and VP2 genes [12]. The complete polyprotein sequence was determined for one of our Canadian Saffold viruses (Can112051-06) (GenBank accession number AM922293). Subsequently, another Saffold virus strain (UC1) was detected in respiratory secretions of a child with influenza-like illness [13] and its complete genomic sequence has also been published (GenBank accession number YP_001949875). The Saffold virus is the first human virus part of the cardiovirus genus which contains two animal species i.e encephalomyocarditis virus species (mengovirus and EMCV) and Theilovirus species (TMEV, Theiler-like virus and Vilyuisk virus) [14]. Phylogenetic analyses suggest that Saffold viruses qualify as divergent members of the Theilovirus species [15]. Comparaison of whole polyprotein sequences (2293 a.a) shows that our Canadian prototype isolate is more related to the UC1 Saffold strain than to the prototype OSV Saffold strain from California (USA) sharing respectively 99.1% and 91.2% amino acid (a.a.) identities with these two strains.

**Figure 1.**
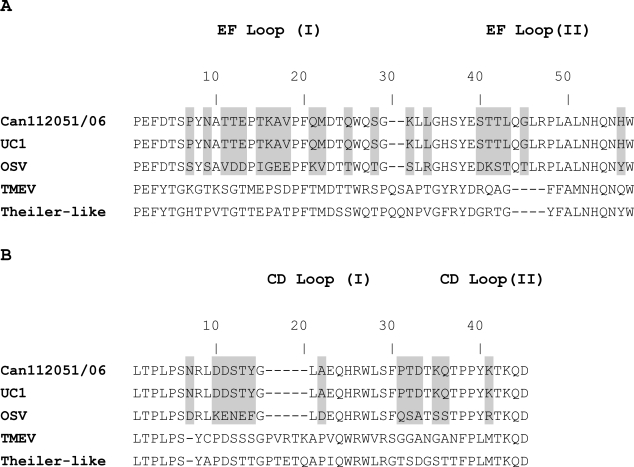
Amino acid sequences of the EF loop (part of the VP2 protein) (A) and the CD loop (part of the VP1 protein) (B) of a Canadian cardiovirus (Can112051-06, GenBank accession number AM922293), other cardioviruses including the original Saffold virus strain (OSV, accession number EF165067) and the UC1 strain (accession number YP_001949875), Theiler’s murine encephalomyelitis virus (TMEV, accession number NP_040350) and Theiler’s-like virus (accession number BAC58035). Amino acid differences between Can112051, UC1 and OSV strains are shaded.

The viral surface of cardioviruses contains four small loops, two of them are part of the EF loop structure within the VP2 protein whereas the two others are part of the CD loop structure within the VP1 protein. The EF loop structure of Can112051-06 strain has 100% and 61.8% a.a. identity with that of UC1 and OSV, respectively (Figure 1A). Similarly, the CD loop structures of Can112051-06 and UC1 are identical and share 67.5% a.a. identity with that of OSV (Figure 1B). Since the EF and CD loop structures are exposed on the viral surface of cardioviruses, they may constitute important sites for recognition by neutralizing antibodies [16]. A possibility thus exists that Can112051-06 and UC1 strains might belong to the same serotype, which could be different than the one represented by OSV. However, further serological studies are needed to verify this hypothesis.

Only 6/17 cases of this study were successfully identified using the SISPA method. Of note, most of the identified viruses (5/6) belong to the Picornaviridae family, suggesting that the primers used for the cDNA synthesis and PCR, although randomly selected, are likely to be more suitable for the identification of picornaviruses. It would be interesting to evaluate the same SISPA approach by using different primers for identification of the remaining 11 cases of this study. The ability of the SISPA method to identify viruses directly from clinical specimens also remains to be investigated although this method should not be viewed as a primary laboratory detection method such as antigen assays, cell culture or PCR-based approaches. Obviously, the sensitivity of virus-specific RT-PCR tests is expected to be significantly higher than SISPA either by using cell culture supernatants or clinical specimens. Therefore, judicious use of such specific tests for viral identification at the family/genus level could decrease the need for more generic methods such as SISPA. Nevertheless, all specific PCR tests could not be performed in the routine clinical setting considering the large number of described respiratory viruses and yet-to-discover pathogens.

In addition to the SISPA method which can be performed in most clinical laboratories, additional strategies requiring specific equipments such as high throughput pyrosequencing [17] and the pan-viral microarray (Virochip) [18] have also proven to be interesting alternatives for the diagnosis of viruses that could not be identified by conventional assays.

## Experimental Section

A panel of 17 clinical respiratory samples collected between 2003–2006 were selected for this study. Identification of viruses from these samples was first attempted by using cell culture-based techniques that consisted of inoculating a panel of cell lines including human laryngeal carcinoma (Hep-2), human foreskin fibroblast, Vero (African green monkey kidney), Mink lung, human lung adenocarcinoma (A-549), human rhabdomyosarcoma (RD), transformed human kidney (293), human colon adenocarcinoma (HT-29), Madin Darby canine kidney (MDCK) and rhesus monkey kidney (LLC-MK2) cells. The presence of CPE or a positive hemadsorption test prompted the performance of immunofluorescent assays for enteroviruses (Pan-Enterovirus Blend kit; Light Diagnostics, Levingston, UK), influenza A and B viruses, parainfluenza viruses 1–4 and adenoviruses (Bartels; Trinity Biotech, Carlsbad, CA), measles and mumps viruses (Chemicon; Billerica, MA), and respiratory syncytial virus (RSV) (Meridian Diagnostics; Cincinnati, OH). In addition, nucleic acids from these 17 samples were obtained using the QIAamp Viral RNA kit (Qiagen, Mississauga, Ontario, Canada) and tested by multiplex real-time RT-PCR assay for the presence of common respiratory viruses (influenza A and B viruses, RSV and human metapneumovirus) [17].

All 17 samples were negative for the above tests despite producing reproducible CPE in LLC-MK2 cells. The supernatants of infected cells were filtered through a 0.22 μm filter and treated with DNase I (Roche Diagnostics; Mannheim, Germany). DNA and RNA were separately extracted using the QIAamp Blood Minikit and QIAamp Viral RNA kit (Qiagen; Mississauga, Ontario, Canada), respectively. Nucleic acids were then used in the SISPA method which was performed as previously described [4]. For RNA virus detection, 10 μL of extracted RNA were incubated with 2 μL (10 μM) of the random SISPA-A primer (5′-GTTCCCAGTCACGATCNNNNNN-3′) for 5 min at 650C, and chilled on ice. Then a 8.7 μL reaction mix containing 4 μL of 5x First-Strand buffer (Invitrogen; Carlsbad, CA), 2 μL of 100 mM DTT, a 2-μL solution containing each dNTP at 5 mM, 0.2 μL of RNase inhibitor (Promega; Madison, WI) and 0.5 μL (100 units) of SuperScriptII reverse transcriptase (Invitrogen; Carlsbad, CA) was added. The reaction was incubated at 25°C for 10 min and at 42°C for 50 min. After a denaturation step of 3 min at 94°C and chilling on ice, 2.5 units (0.5 μL) of 3′-5′ exo-Klenow DNA polymerase (New England Biolabs) were added and the reaction was incubated at 37°C for 1 h. The enzyme was inactivated at 75°C for 10 min and, finally, 5 μL of the resulting double-stranded cDNA were used for PCR amplification using the SISPA-B primer (5′-GTTCCCAGTCACG ATC-3′) and the PFU *Turbo* DNA polymerase (Stratagene, La Jolla, CA) following the manufacutrer’s recommendations. PCR conditions included a denaturation step of 10 min at 94°C, followed by 40 cycles of amplification (94°C for 1 min, 65°C for 1 min and 72°C for 2 min). After a 1.2% agarose gel electrophoresis, PCR products of 600–1,600 bp were purified using the gel-extraction kit (Sigma-Aldrich; Oakville, ON) and cloned using the CloneJET PCR Cloning Kit (Fermentas; Burlington, ON). Two recombinant plasmids for each insert were sequenced before comparison with GenBank database using the tBLASTx program. For DNA virus detection, we used the same conditions as previously described [4].

## Conclusions

Our study highlights the limitation of conventional virological diagnostic methods as well as selected molecular PCR assays for identification of emerging or non-conventional viruses and confirms the usefulness of a new method based on random PCR amplification i.e. SISPA. Using this relatively simple method, we were able to identify rare (influenza C) or emerging (HPeV-3, cardiovirus Saffold) viruses in 6/17 (35%) respiratory samples associated with uncharacterized CPE in cell culture.

## Figures and Tables

**Table 1.** Identification of viruses by using the SISPA method.

**Isolate/year**	**Specimen**	**Virus**	**Time to CPE**

Can90775/2003	NPA	Influenza C	19 days
Can101516/2005	BAL	HPeV-3	6 days
Can101519/2005	BAL	HPeV-3	6 days
Can112051/2006	NPA	Cardiovirus Saffold	4 days
Can116603/2006	NPA	Cardiovirus Saffold	4 days
Can116604/2006	NPA	Cardiovirus Saffold	4 days

NPA, nasopharyngeal aspirate; BAL, bronchoalveolar lavage; HPeV, human parechovirus; CPE, cytopathic effects.
